# Studying Music During the Coronavirus Pandemic: Conditions of Studying and Health-Related Challenges

**DOI:** 10.3389/fpsyg.2021.651393

**Published:** 2021-03-08

**Authors:** Magdalena Rosset, Eva Baumann, Eckart Altenmüller

**Affiliations:** ^1^Department of Journalism and Communication Research, Hanover University of Music, Drama and Media, Hanover, Germany; ^2^Institute of Music Physiology and Musicians’ Medicine, Hanover University of Music, Drama and Media, Hanover, Germany

**Keywords:** coronavirus, pandemic, musicians’ health, music college, performance, music education, music students

## Abstract

**Objective:**

The coronavirus pandemic affects all areas of life. Performing arts and music studies have also experienced considerable changes, with university closures and a fluctuating return to normal and more limited operations. Prior studies detail the impact of the pandemic on college students, but we do not yet know what specific consequences it has for music students. The aim of this study is to examine the impact of the coronavirus pandemic on music students’ health, practicing behavior, and everyday life.

**Methods:**

In July 2020, we conducted an online survey of 80 students enrolled in performance and music education programs at a German music university and compared these data with data from a survey of 75 students at the same music university carried out in summer 2019.

**Results:**

The survey revealed that the coronavirus pandemic led to a decrease in practicing hours and an increase of stressful thoughts and feelings. Students were mostly satisfied with Corona-measures taken by the university. Of analyzed determinants, only general fear of health problems was identified as a significant predictor of mental health status. Mental health status did not significantly differ between students surveyed in 2019 and 2020.

**Conclusion:**

Knowledge about the specific challenges the pandemic poses for music students can help conservatories to better respond to the needs of their students. Specifically, this study will inform future measures supporting music students in coping with difficult situations like a pandemic.

## Introduction

In addition to the general stressors experienced by many during the coronavirus pandemic (e.g., social isolation, potential loss of earnings, or fear of infection), music students had to deal with specific stressors. The quality of online teaching is reduced compared to one-to-one teacher-student-contact in instrumental and singing classes due to reduced fidelity of sound transmission and an inability of teachers to holistically perceive body movements, posture, and performance emotions. Additionally, normal daily routines have been disrupted, such as practicing in the university’s rehearsal rooms or socializing with classmates. Ensemble practice, chamber music, and choir singing are restricted, and public concerts and performances are impossible. Intense goal directed practicing and mentoring during music studies is absolutely necessary to attain artistic goals and win auditions or competitions (which are almost all canceled), meaning that students fear the “loss” of a critical year of development. Financial issues are an additional cause of worries (e.g., Sahu, 2020), as well as a general societal and government neglect of cultural interests (Botstein, 2020) leading students to perhaps increasingly question the meaningfulness of studying music.

Studies in the general population show the negative effects of the coronavirus pandemic on mental health and wellbeing (e.g., Huang and Zhao, 2020; Sønderskov et al., 2020; Wang C. et al., 2020; Xiong et al., 2020). Being female, younger age, a student, and having negative coping styles is associated with more negative psychological effects, while a perceived lower risk of infection is associated with lesser negative effects (Huang and Zhao, 2020; Liu et al., 2020; Wang C. et al., 2020; Wang H. et al., 2020; Xiong et al., 2020). Higher education students are particularly vulnerable to mental health problems compared to other populations (Auerbach et al., 2016), a trend which has continued during the coronavirus pandemic. During the pandemic, higher education students have been shown to experience higher levels of stress, anxiety, loneliness, and symptoms of depression than before the pandemic (Elmer et al., 2020; Huckins et al., 2020). Studies emphasize the impact of the pandemic on college students, demonstrating psychological difficulties, increased symptoms of depression and anxiety, and a self-reported decline in mental health (Cao et al., 2020; Huckins et al., 2020; Odriozola-González et al., 2020). This might be due to college students’ young age, with studies showing that younger people are psychologically more affected by the pandemic than older people (Huang and Zhao, 2020; Xiong et al., 2020). Furthermore, findings identified the mental health of female university students as more likely to be affected than male students (Elmer et al., 2020). In addition, concerns about family and friends are associated with a worsening of mental health status (Elmer et al., 2020).

As reviewed above, there is much information available how the pandemic is affecting the general public and college students. However, we do not yet know the specific consequences for music students. To address this research gap, the aim of this study was to examine the impact of the coronavirus pandemic on music students’ physical and mental health status, their practicing behavior, and their everyday life. Our first research question was:

RQ1: What is the situation of music students during the coronavirus pandemic with respect to a) infection status, belonging to a risk group or caring for someone belonging to a risk group, b) assessment of the probability and risk of a SARS-CoV-2 infection, c) playing-related pain, d) daily practicing time, e) mental health status, f) a possible loss of earnings and financial distress, g) giving digital music lessons or playing virtual concerts, and h) assessment of the university’s measures and handling of the coronavirus pandemic.

Since the curriculum and requirements of performance classes differ from those of music education programs, we proposed a second research question:

RQ2: Are there differences between performance majors and music education majors regarding the aspects under investigation?

Two more research questions were focused on a closer examination of the music students’ mental health status. The third one referred to a temporal comparison of the students’ mental health status, while the fourth addressed determinants of their mental health status:

RQ3: How does the perceived mental health status differ in students interviewed during the pandemic and students interviewed in 2019?
RQ4: Which factors influence music students’ mental health during the coronavirus pandemic?

To identify factors of the coronavirus pandemic influencing the mental health status of music students, determinants that have been identified in studies of the general public and college students were considered (age, gender, risk perceptions, concern for loved ones, and coping strategies). To clarify the specific challenges of studying music during the pandemic, we also included specific characteristics of studying music (performance major vs music education major, change in playing-related pain during the pandemic, and change in practicing time during the pandemic). Furthermore, we wanted to explore if the measures taken by the university during the coronavirus pandemic had an influence on mental health status. Since financial concerns have been shown to increase symptoms of depression, anxiety, and stress in college students (Beiter et al., 2015), experiences of financial distress due to the pandemic were analyzed as an additional influencing factor. Finally, as the coronavirus pandemic could be more worrying for those who generally are more concerned about their health, we considered the impact of general fear of health problems on student mental health.

## Methods

### Dealing With the Coronavirus Pandemic at the University

During the coronavirus pandemic, the presidential board of our university decided to close the university completely from March 15 until May 7, adhering to the specific corona-regulations of the state of Lower Saxony in Germany. From May 8 on, the university opened partially, allowing limited access to practice rooms under strict hygiene regulations and highly restricted time schedules for all students (2 to 4 h a week), as well as social distance, mask wearing, and disinfection rules. Face-to-face lessons were allowed, however more than half of teachers and professors had to stay in their home countries due to international mobility restrictions or their status as “persons at risk” according to the German coronavirus regulations. These teachers were asked to offer lessons via video conferencing.

### Participants

Between July 6 and July 27, 2020, an online-survey was distributed via e-mail to all bachelor students enrolled in performance training and music education training (basic population *N* = 157) at a German music college. The sample consisted of *n* = 80 students (response rate: 51%). Performance training comprises the programs musical performance, pianoforte, jazz and jazz-related music, and popular music. Music education training comprises the programs music performance and education and an interdisciplinary bachelor degree. Usually, students in music education training practice their main instrument less and are more involved in multi-instrumental practice, choir conducting, and theoretical pedagogical seminars. The survey was available in German and English, with 75 respondents choosing the German version (94%) and 5 respondents the English version (6%). At the beginning of the online survey, the subject and purpose of the study and the voluntary nature of participation was explained, the anonymity and confidential handling of the data was assured, and the participants were informed that they could withdraw their consent to participate in the survey at any time. The participants gave their informed consent to take part in the study prior to entering the main survey. The study is approved by the joint ethics committee of the Leibniz University Hannover and the Hanover University of Music, Drama and Media (EV-LUH 9/2017). We furthermore adhere to the ethics regulations of our university according to the guidelines of the German Research Foundation and the Declaration of Helsinki. Participants were compensated for their time with 20 euros. Sample characteristics are provided in Table 1.

**TABLE 1:** Sample characteristics (performance majors and music education majors).

	2020						2019	
	Performance majors (*n* = 26, 33%)		Music education majors (*n* = 54, 68%)		Total (*n* = 80)		Total (*n* = 75)	
	*n/M*	%/*SD (min.; max.)*	*n/M*	%/*SD (min.; max.)*	*n/M*	%/*SD (min.; max.)*	*n/M*	%/*SD (min.; max.)*
**Field of study**								
Performance majors							20	27%
Music education majors							55	73%
**Gender**								
Female	12	46%	30	56%	42	53%	42	56%
Male	14	54%	24	44%	38	48%	33	44%
**Age**	20.62	*SD* = 2.33 (*min* = 17; *max* = 28)	20.96	*SD* = 2.14 (*min* = 18; *max* = 30)	20.85	*SD* = 2.19 (*min* = 17; *max* = 30)	21.11	*SD* = 2.93 (*min* = 17; *max* = 33)
**Semester**	2.58	*SD* = 0.95 (*min* = 1; *max* = 4)	2.93	*SD* = 1.08 (*min* = 2; *max* = 6)	2.81	*SD* = 1.04 (*min* = 1; *max* = 6)	2.73	*SD* = 1.04 (*min* = 2; *max* = 6)
First citizenship***								
German	15	58%	51	94%	66	83%	64	85%
Other	11	42%	3	6%	14	18%	11	15%
**Main instrument**								
String instruments (without plucking instruments)	6	23%	15	28%	21	26%	9	12%
Wind instruments	8	31%	13	24%	21	26%	20	27%
Piano, organ	3	12%	13	24%	16	20%	23	31%
Plucking instruments	2	8%	5	9%	7	9%	5	7%
Voice	3	12%	4	7%	7	9%	13	17%
Percussion instruments	4	15%	2	4%	6	8%	3	4%
Composition, music theory	0	0%	2	4%	2	3%	2	3%

2020: n = 80 (n_performance major_ = 26; n_music__education major_ = 54). Differences between performance majors and music education majors assessed using chi^2^-tests and independent samples t-tests: ***p ≤ 0.001, **p ≤ 0.01, *p ≤ 0.05. First citizenship: χ ^2^(8, n = 80) = 27.16, Cramer’s V = 0.58, p ≤ 0.001. Others: n.s. 2019: n = 75, all comparisons between the 2019 and 2020 data: n.s.

Comparative data was collected between June 24 and July 21, 2019. The comparative sample consisted of *n* = 75 students (response rate: 60%). None of the respondents took part in both surveys. The procedure of data collection was identical in 2019 and 2020.

### Measures

At our university, we have conducted an annual online survey of the music students’ wellbeing and health since 2017. These data will be published separately. In summer 2020, we added questions related to the new study conditions resulting from the coronavirus pandemic and related regulations (see above). The survey asked if students were *infected with SARS-CoV-2* (either confirmed by a test or assumed infection not confirmed by a test), if they *belonged to a group with a higher risk* for a severe course and poor outcomes from COVID-19, or if they *lived with someone or took care for someone belonging to a risk group* (as proxy for concern for loved ones). Further, the questionnaire assessed on five-point Likert-type scales the *perceived likelihood of getting infected* (1 “extremely unlikely” to 5 “extremely likely”) and the *perceived severity of an infection* (1 “not severe” to 5 “very severe”).

The questionnaire also included items on *changes regarding physical pain* while playing (1 “pain is much weaker than before the coronavirus pandemic,” 3 “no change, and 5 “pain is much stronger than before the coronavirus pandemic”) and *mental state* (1 “I had much less stressful thoughts and feelings during the coronavirus pandemic than before,” 3 “No change,” and 5 “I had much more stressful thoughts and feelings during the coronavirus pandemic than before”). Respondents were also asked to indicate on a five-point Likert-type scale (1 “none at all” to 5 “very much”) their perceived stress over the past week in eight different domains (e.g., “feeling fearful”) to assess their *mental health status*. The items were taken from the eight-item symptom checklist (SCL-8), a short form of the SCL-25 measuring symptoms of depression and anxiety (Tambs and Røysamb, 2014). The scale showed high internal consistency in this study (α = 0.88) and the items were combined into a mean index.

In addition, students were asked *how many hours they practiced daily on average* before the pandemic and if there was a *change in their daily practicing hours* during the pandemic. Respondents indicating such a change were asked to clarify their answer on a five-point scale (1 “I practiced a lot more during the coronavirus pandemic than before the pandemic,” 3 “No change,”, and 5 “I practiced much less during the coronavirus pandemic than before the pandemic”) and to mark *reasons for an increase* (e.g., “With practicing, I distracted myself from other worries.”) or *decrease in practicing time* (e.g., “I did not have access to a rehearsal room.”). In an open-ended question, respondents had the opportunity to give other reasons for an increase or decrease in practicing hours.

Further, respondents were asked if they played *virtual concerts* and, if so, which tools they used and if they shared concerts on digital platforms (e.g., YouTube) or live streamed them (using e.g., Instagram). Respondents were also asked if they gave *virtual music lessons* during the pandemic. Other items assessed a possible *loss of earnings* due to concert cancelations or loss of side jobs, and if the pandemic caused *financial distress*.

The perception of the *university’s measures taken during the pandemic and handling of the pandemic* was measured on a five-point Likert-type scale (1 “strongly disagree” to 5 “strongly agree”) using nine items (e.g., “The university provides good information about the risk of infection in the university context.”). The scale showed high internal consistency (α = 0.83) and the items were combined into a mean index.

Further, general (not coronavirus-specific) *fear of health problems* was assessed by asking the respondents’ response to one item (“I am scared of having health problems.”) on a five-point Likert-type scale (1 “doesn’t apply at all” to 5 “applies completely”).

Finally, items regarding different *coping strategies* (*positive thinking*: e.g., “I tell myself that stress and pressure also have positive effects”; *active coping*: e.g., “I do everything to prevent stress in the first place.”; *social support*: e.g., “When I am stressed or under pressure, I find support from my partner or a good friend.”; *faith*: e.g., “When I am stressed or under pressure, I find relief in my faith.”; *alcohol and cigarettes*: e.g., “When I am stressed or under pressure I relax with a glass of wine or beer in the evening.”) were included using the Stress and Coping Inventory (SCI; Satow, 2012). All five coping strategies were assessed with four items using a five-point Likert-type scale (1 “doesn’t apply at all” to 5 “applies completely”). The five subscales showed satisfactory internal consistencies (positive thinking: α = 0.64; active coping: α = 0.75; social support: α = 0.81; faith: α = 0.77; alcohol and cigarettes: α = 0.73) and the items for each coping strategy were combined into a mean index.

### Data Analysis

Data were analyzed using SPSS (v. 27). Descriptive analyses were conducted for the sample using frequencies and percentages for categorical data and means (*M*) and standard deviations (*SD*) for numeric data. Differences between the two groups (performance majors vs music education majors) were assessed using chi-square tests and independent samples *t*-tests. Differences in music students’ mental health status between 2019 and 2020 were evaluated using an independent samples *t*-test.

To explore the impact of different determinants of mental health status, a hierarchical linear regression analysis was performed. Change in *R*^2^ (Δ*R*^2^) was computed to determine the relative contribution of each model. In the first block, age, gender, and degree focus (performance majors vs music education majors) were included to control for the effect of these variables. In the second block, the COVID-19-related items were estimated, since previous studies had shown that coronavirus-related factors can influence mental health status (living with/caring for someone belonging to a risk group, perceived likelihood of getting infected with SARS-CoV-2, perceived severity of an infection, change in playing-related pain during the pandemic compared to before, financial distress due to the pandemic, assessment of change in daily practicing hours, perception of the university’s measures taken during the pandemic and handling of the pandemic). In the third block, fear of health problems was included, since we assumed that the coronavirus pandemic could be more worrying for those who generally are more concerned about their health. In the fourth block, five different coping strategies (social support, positive thinking, active coping, faith, alcohol, and cigarettes) were entered into the regression model, since previous studies had shown an influence of coping strategies on mental health in the general population. However, we wanted to analyze the influence of specific forms of coping strategies which were not yet accounted for in previous research on students’ mental health during the coronavirus pandemic. Thus, we included these variables in the last model.

## Results

### Sample Characteristics

Of the overall sample, 26 respondents were performance majors (33%) and 54 were music education majors (68%; see Table 1). There were slightly more female students in the sample (53%). Students in the sample were on average 20.85 years old (*SD* = 2.19, range: 17 to 30) and had a German citizenship (83%). Most of the students were in their second semester (56%), followed by students in their fourth semester (38%). In the sample, 21 (26%) students played wind instruments as main instrument, 21 (26%) played string instruments, 16 (20%) played keyboard instruments, 7 (9%) sang, 7 (9%) played plucked instruments, 6 (8%) played percussion instruments, and 2 (3%) of the students were enrolled in music theory or composition. Concerning sample characteristics, students enrolled in performance training differed from students in music education training only regarding their first citizenship, with performance majors having a higher rate of students with another first citizenship than German than music education majors (see Table 1). Sample characteristics of the data collected in 2019 are shown in Table 1. Chi-square tests showed no significant differences between the data collected in 2019 and 2020 regarding gender, first citizenship, studying performance training or music education training, and main instrument. Independent samples t-tests showed no significant differences between the participants in 2019 and in 2020 concerning age and semester. There were no significant differences regarding sample characteristics between the data collected in 2019 and in 2020.

### Descriptive Results

To answer the first research question (RQ1), we looked at descriptive results. None of the respondents had a confirmed infection with SARS-CoV-2 and only one reported having had an infection which had not been confirmed by a test (see Table 2). Most of the interviewed students did not belong to group at a higher risk for a severe course of COVID-19, but 23% lived with someone or cared for someone belonging to a risk group. The respondents estimated their probability of getting infected with SARS-CoV-2 (*M* = 2.54) and the severity of a possible infection for themselves (*M* = 2.49) as rather low.

**TABLE 2:** Descriptive results (performance majors and music education majors).

	Performance majors (*n* = 26, 33%)		Music education majors (*n* = 54, 68%)		Total (*n* = 80)	
	*n/M*	%/*SD (min.; max.)*	*n/M*	%/*SD (min.; max.)*	*n/M*	%/*SD (min.; max.)*
**SARS-CoV-2 infection**						
Yes, the infection was confirmed by a test	0	0%	0	0%	0	0%
Yes, I think so, but the infection was not confirmed by a test	1	4%	0	0%	1	1%
No	18	69%	44	82%	62	78%
Don’t know	7	27%	10	19%	17	21%
**Belonging to a risk group**						
Yes	0	0%	1	2%	1	1%
No	22	85%	51	94%	73	91%
Don’t know	4	15%	2	4%	6	8%
**Living with/caring for someone belonging to a risk group**						
Yes	6	23%	12	22%	18	23%
No	20	77%	41	76%	61	76%
Don’t know	0	0%	1	2%	1	1%
Perceived likelihood of getting infected with SARS-CoV-2	2.60	*SD* = 0.91 (*min* = 1; *max* = 5)	2.52	*SD* = 0.86 (*min* = 1; *max* = 5)	2.54	*SD* = 0.87 (*min* = 1; *max* = 5)
Perceived severity of an infection**	2.92	*SD* = 1.15 (*min* = 1; *max* = 5)	2.30	*SD* = 0.82 (*min* = 1; *max* = 4)	2.49	*SD* = 0.97 (*min* = 1; *max* = 5)
Change in playing-related pain during the pandemic compared to before the pandemic (1 = less pain; 3 = no change; 5 = more more)	2.76	*SD* = 0.83 (*min* = 1; *max* = 4)	2.96	*SD* = 0.76 (*min* = 1; *max* = 5)	2.89	*SD* = 0.78 (*min* = 1; *max* = 5)
Change in mental state during the pandemic compared to before the pandemic (1 = fewer worrying thoughts and feelings; 3 = no change; 5 = more worrying thoughts and feelings)	3.92	*SD* = 0.98 (*min* = 1; *max* = 5)	3.65	*SD* = 0.78 (*min* = 2; *max* = 5)	3.74	*SD* = 0.85 (*min* = 1; *max* = 5)
Symptoms of depression and anxiety (SCL-8) (1 = higher mental health status, 5 = lower mental health status)	2.85	*SD* = 0.91 (*min* = 1; *max* = 5)	2.70	*SD* = 0.93 (*min* = 1; *max* = 5)	2.75	*SD* = 0.92 (*min* = 1; *max* = 5)
Average daily practicing hours before the pandemic*	3.23	*SD* = 1.51 (*min* = 0; *max* = 6)	2.57	*SD* = 1.30 (*min* = 0; *max* = 6)	2.79	*SD* = 1.39 (*min* = 0; *max* = 6)
**Change in daily practicing hours during the pandemic compared to before the pandemic**						
Yes	21	81%	40	74%	61	76%
No	5	19%	14	26%	19	24%
Assessment of change in daily practicing hours (1 = more; 3 = no change; 5 = less)	3.12	*SD* = 1.34 (*min* = 1; *max* = 5)	3.33	*SD* = 1.37 (*min* = 1; *max* = 5)	3.26	*SD* = 1.36 (*min* = 1; *max* = 5)
Increase in daily practicing hours	10	39%	15	28%	25	31%
**Reasons for increase in daily practicing hours (multiple response)**						
I had more time than before the pandemic.	7	27%	14	26%	21	26%
I was able to practice well at home.	8	31%	11	20%	19	24%
I always had my instrument within reach at home.	7	27%	10	19%	17	21%
I was able to practice independently of the lessons.	5	19%	10	19%	15	19%
I was able to concentrate better.	4	15%	4	7%	8	10%
I could motivate myself better.	3	12%	4	7%	7	9%
With practicing, I distracted myself from other worries.	2	8%	1	2%	3	4%
Other (open-ended)	1	4%	3	6%	4	5%
Decrease in daily practicing hours	11	22%	25	46%	36	45%
**Reasons for decrease in daily practicing hours (multiple response)**						
I was less motivated.	9	35%	20	37%	29	36%
I did not have access to a rehearsal room.	7	27%	14	26%	21	26%
It was more difficult for me to concentrate.	6	23%	15	28%	21	26%
I had too many other worries.	4	14%	10	19%	14	18%
I did not have any classes to practice for.	5	19%	6	11%	11	14%
I had no access to an instrument.	4	15%	6	11%	10	13%
I had less time than before the pandemic.*	0	0%	7	13%	7	9%
Other (open ended)	4	15%	4	7%	8	10%
**Loss of earnings due to not playing concerts**						
Yes	13	50%	22	41%	35	44%
No	13	50%	32	59%	45	56%
Only students having side-jobs: Loss of earnings due to not being able to work a side-job**	*n* = 3		*n* = 32		*n* = 35	
Yes	0	0%	24	75%	24	69%
No	3	100%	8	25%	11	31%
**Financial distress due to the pandemic**						
Yes	5	19%	10	19%	15	19%
No	19	73%	38	70%	57	71%
No answer/not applicable	2	8%	6	11%	8	10%
Only students giving music lessons: **Providing digital music lessons**	*n* = 2		*n* = 21		*n* = 23	
Yes	2	100%	11	52%	13	57%
No	0	0%	10	48%	10	44%
**Digital performance of concerts, competitions and/or auditions**						
Yes	3	12%	9	17%	12	15%
No	23	89%	45	83%	68	85%
Perception of university’s measures taken during the pandemic and handling of the pandemic	3.64	*SD* = 0.72 (*min* = 2; *max* = 5)	3.60	*SD* = 0.69 (*min* = 2; *max* = 5)	3.62	*SD* = 0.69 (*min* = 2; *max* = 5)

n = 80 (n_performance major_ = 26; n_music education ma__j__or_ = 54). Differences between performance majors and music education majors assessed using chi^2^-tests and independent samples t-tests: ***p ≤ 0.001, **p ≤ 0.01, *p ≤ 0.05. Assessment of severity of an infection: T(77) = 2.76, p ≤ 0.01. Average daily practicing hours before the pandemic: T(78) = 2.01, p ≤ 0.05; reasons for decrease in daily practicing hours: I had less time than before the pandemic: χ^2^(1, n = 36) = 3.82, φ = 0.33, p ≤ 0.05. Loss of earnings due to not being able to work a side-job: χ^2^(1, n = 35) = 7.16, φ = −0.45, p ≤ 0.01. Others: n.s.

Concerning playing-related factors, respondents reported rather no change in playing-related pain during the coronavirus pandemic (*M* = 2.89), which might be due to a change in practicing time. Before the pandemic, the students practiced on average 2.79 h a day. Seventy-six% indicated a change in practicing hours during the pandemic. Thirty-one% of all respondents reported an increase in daily practicing hours, while 45% reported a decrease. The most frequently mentioned reasons for an increase in daily practicing were that students had more time at hand during the pandemic, that they could practice well and always had their instrument within reach at home. Most frequent reasons for a decrease in daily practicing were a loss of motivation and concentration as well as a lack of access to a rehearsal room. Some students also pointed to mental health problems as a reason for less practicing.

Mental health issues become also evident in the change in students’ self-assessment of their mental state: Comparing their mental state during the coronavirus pandemic to the time before the pandemic, respondents reported rather more stressful thoughts and feelings (*M* = 3.74).

Regarding financial issues during the coronavirus pandemic, several respondents reported a loss of earnings due to the cancelation of concerts (44%; 35 of the 80 participants) or because they could not work in their side-job (69%; 24 of the 35 students working a side-job). Nevertheless, only 19% of all participants indicated that they experienced financial distress due to the pandemic. Concerning a switch to digital channels, of 23 students that gave music lessons, 57% reported that they provided digital music lessons during the coronavirus pandemic. Only 15% of the respondents played digital concerts during the pandemic. Most of those who did, reported in an open-ended question that they used Zoom.

When asked about their assessment of the university’s measures and handling of the pandemic, respondents seemed rather satisfied (*M* = 3.63). They perceived that the university tried to protect students from the virus, offered care to the students, was open about its strategy and provided good information about infection risks in the university context, was competent in dealing with the virus, and honest about how to deal with the pandemic. Further, they perceived that the university could help protect them from the virus and that the university’s measures were appropriate and in their personal interest.

### Differences Between Performance Majors and Music Education Majors

Since the study requirements for students enrolled in performance classes and those in music education training differ, we looked at differences between these two groups across analyzed variables (RQ2). There were significant differences in the estimated severity of an infection (*T*(77) = 2.76, *p* ≤ 0.01; see Table 2) – performance majors perceived that an infection would be slightly more severe (*M* = 2.92) than music education majors (*M* = 2.30). Further, performance majors reported significantly higher practicing hours before the pandemic (*M* = 3.23) than music education majors (*M* = 2.57; *T*(78) = 2.01, *p* ≤ 0.05). Performance majors and music education majors also differed in one of the inquired reasons for a decrease in daily practicing hours: Some of the music education majors reported that they practiced less during the pandemic since they had less time than before the pandemic (*n* = 7; 28%), while none of the performance majors gave this response [χ^2^(1, *n* = 36) = 3.82, φ = 0.33, *p* ≤ 0.05]. Finally, there were significant differences between the two groups regarding the loss of earning due to not being able to work a side-job [χ^2^(1, *n* = 35) = 7.16, φ = −0.45, *p* ≤ 0.01]: while this did not apply to any of the performance majors, it was true for some of the music education majors (*n* = 24; 75%).

### Comparison of Music Students’ Perceived Mental Health Status During the Pandemic and Before the Pandemic

To further assess music students’ mental health status, we compared the current data with comparable data collected in 2019 (RQ3). There were no significant differences regarding self-assessed symptoms of depression and anxiety between students who were interviewed during the pandemic 2020 (*M* = 2.75, *SD* = 0.92) and students who were interviewed a year earlier (*M* = 2.68, *SD* = 0.89) (*T*(153) = −0.48, n.s.).

### Determinants of Mental Health Status

A hierarchical linear regression model assessed the influence of possible determinants of the students’ mental health status during the coronavirus pandemic (RQ4; see Table 3). All predictors accounted for 25% of the overall variance in mental health status [*R*^2^*_*adj.*_* = 0.25, *F*(16,51) = 2.43, *p* ≤ 0.01]. The first model did not lead to a significant explanation of total variance (*R*^2^*_*adj.*_* = 0.05). The only significant predictor in the first model was gender (β = −0.27), with female students reporting a lower mental health status. In the second model, the inclusion of the COVID-19-related items did also not account for a significant increase in *R*^2^ (Δ*R*^2^ = 0.16; *R*^2^*_*adj.*_* = 0.12). Of the coronavirus-related items, only financial distress due to the pandemic (β = 0.25) was a significant predictor of the mental health status, with students who were in financial distress experiencing more health problems. In model 3, the inclusion of general fear of health problems lead to a significant increase of explained variance (Δ*R*^2^ = 0.10; *R*^2^*_*adj.*_* = 0.22), with students who reported a higher fear of health problems showing a decreased mental health status (β = 0.35). Including fear of health problems lead to a decrease of the influence of financial distress due to the pandemic, which no longer had a significant effect on mental health status in the third model. The inclusion of different coping strategies in the final model lead to only a small and non-significant increase in explained variance (Δ*R*^2^ = 0.08; *R*^2^*_*adj.*_* = 0.25), with none of the included items being significant predictors of mental health status. In the final model, only fear of health problems showed a significant influence on mental health status (β = 0.35), whereas gender had no longer a significant effect when controlling for all other included variables.

**TABLE 3:** Predictors of mental health status.

	Mental health status					
	Model 1	Model 2	Model 3	Model 4		
		
Variable	*b*	*b*	*b*	*b*	95% CI	β
Age	0.06	0.06	0.03	0.03	[−0.07, 0.12]	0.07
Gender	−0.48*	−0.50*	−0.52**	−0.36	[−0.83, 0.12]	−0.20
Performance major/music education major	−0.06	−0.08	−0.06	−0.03	[−0.48, 0.42]	-0.02
Living with/caring for someone belonging to a risk group		−0.25	−0.28	−0.27	[−0.77, 0.22]	−0.13
Perceived likelihood of getting infected with SARS-CoV-2		−0.10	−0.11	−0.15	[−0.37, 0.08]	−0.15
Perceived severity of an infection		0.07	0.09	−0.01	[−0.27, 0.25]	-0.01
Change in playing-related pain during the pandemic compared to before the pandemic		0.25	0.13	0.21	[−0.09, 0.50]	0.18
Financial distress due to the pandemic		0.55	0.39	0.34	[−0.21, 0.90]	0.16
Assessment of change in daily practicing hours		0.10	0.09	0.02	[−0.15, 0.18]	0.03
Perception of university’s measures taken during the pandemic and handling of the pandemic		0.09	0.09	0.00	[−0.32, 0.33]	0.00
Fear of health problems			0.26**	0.26***	[0.08, 0.44]	0.35
Social support as coping strategy				−0.25	[−0.53, 0.03]	−0.22
Positive thinking as coping strategy				−0.21	[−0.48, 0.07]	−0.18
Active coping as coping strategy				0.01	[−0.24, 0.27]	0.01
Faith as coping strategy				0.23	[−0.03, 0.50]	0.25
Alcohol and cigarettes as coping strategy				−0.08	[−0.36, 0.21]	−0.07
*R*^2^	0.30	0.50	0.59	0.66		
*R*^2^_*adj.*_	0.05	0.12	0.22	0.25		
Δ*R*^2^		0.16	0.10	0.08		
*F*	2.13	1.93	2.75**	2.43**		

N = 68. Mental health status: 1 = higher mental health status, 5 = lower mental health status. Gender: 0 = female, 1 = male; performance major = 0, music education major = 1. ***p ≤ 0.001, **p ≤ 0.01, *p ≤ 0.05. b = unstandardized regression coefficient, β = standardized regression coefficient, CI = confidence interval. F(16,51) = 2.43, p ≤ 0.01.

## Discussion

There is a growing body of research on the effects of the coronavirus pandemic in the general population and some evidence for university students, yet to our knowledge no studies of music students have been published. Our study addresses this research gap by providing the first insights into the situation of music students during the pandemic. In line with previous research on higher education students in general (Odriozola-González et al., 2020), our results indicate that music students perceive the pandemic as challenging, particularly with regard to their mental health status. For some students, this might also have negatively influenced practicing hours since they reported problems concentrating and motivating themselves. Even though the study curriculum and the demands of the programs of performance majors and music education majors considerably differ, the coronavirus pandemic affected them similarly. It is unsurprising that these two groups differed in their daily practicing hours, with music education majors having more other (e.g., educational) courses and generally not practicing as long as performance majors. Nevertheless, there were no significant differences between the two groups regarding the self-assessed change in practicing hours during the pandemic. Different from earlier studies in the general population (Wang C. et al., 2020) or among university students (Elmer et al., 2020), our study could only identify fear of health problems as a significant predictor of mental health status. Further research is needed to explore the influencing factors of music students’ psychological state and to figure out if influencing patterns are different during crises compared to “normal” semester times.

In contrast to studies comparing data from university students during and before the coronavirus pandemic (Elmer et al., 2020; Huckins et al., 2020), our analysis did not show significant differences in music students’ mental health status during compared to before the pandemic. This could be due to our use of a measure of mental health status designed to assess symptoms of anxiety and depression, symptoms which might be too serious to occur on a short time scale during stressful times. Further, this finding may be due to a ceiling effect. Independent from the coronavirus pandemic, music students suffer particularly from mental distress (e.g., Wristen, 2013), which may have prevented this already large burden from increasing further during the pandemic. Nevertheless, when asked to assess a change in worrying thoughts and feelings during the coronavirus pandemic compared to before the pandemic, respondents who completed the survey in 2020 reported rather more worrying thoughts and feelings than before the pandemic. However, this result is based on participants’ self-assessments and is merely descriptive. Further, there is no data collected before the pandemic for comparison.

Our results have several limitations: 1) The results are based on a relatively small sample size, since we only investigated students from one music university. 2) A relatively low percentage of performance majors participated compared to music education majors. 3.) The sample was self-selected and therefore potentially biased. 4.) The data are cross-sectional and not panel data. Due to these limitations, the generalizability of the results remains questionable. Moreover, at the time of data collection, infection rates in Germany were relatively low. This might have influenced the perceived likelihood of getting infected and other coronavirus-related assessments. Further, several measures were assessed using only one item, with self-reported measures further risking the collection of biased data. Finally, with a relatively high number of included predictors, the regression model is subject to the risk of overfitting. This should be considered when interpreting the results.

Overall, the results provide the first insights into the consequences of the coronavirus pandemic for music students. There is a need to examine the challenges the pandemic poses for music students in more detail, focusing especially on influencing factors and long-term effects. Knowledge about music students’ specific challenges during crises can help music universities to better respond to the needs of their students and inform future measures to help music students cope with difficult situations like a pandemic.

## Data Availability Statement

The raw data supporting the conclusions of this article will be made available by the authors, without undue reservation.

## Ethics Statement

The study was reviewed and approved by the joint ethics committee of the Leibniz University Hannover and the Hanover University of Music, Drama and Media. The participants provided their informed consent to participate in this study.

## Author Contributions

MR designed the questionnaires, performed the survey, analyzed the data, discussed the data, and wrote the manuscript. EB designed the questionnaires, analyzed the data, discussed the data, and wrote the manuscript. EA designed the questionnaires, recruited the students, discussed the data, and wrote the manuscript. All authors contributed to the article and approved the submitted version.

## Conflict of Interest

The authors declare that the research was conducted in the absence of any commercial or financial relationships that could be construed as a potential conflict of interest.

## References

[B1] Auerbach R. P. Alonso J. Axinn W. G. Cuijpers P. Ebert D. D. Green J. G. (2016). Mental disorders among college students in the World Health Organization world mental health surveys. *Psychol. Med.* 46 2955–2970. 10.1017/S0033291716001665 27484622 PMC5129654

[B2] Beiter R. Nash R. McCrady M. Rhoades D. Linscomb M. Clarahan M. (2015). The prevalence and correlates of depression, anxiety, and stress in a sample of college students. *J. Affect. Disord.* 173 90–96. 10.1016/j.jad.2014.10.054 25462401

[B3] Botstein L. (2020). The future of music in America: the challenge of the COVID-19 pandemic. *Musical Q.* 102 351–360. 10.1093/musqtl/gdaa007

[B4] Cao W. Fang Z. Hou G. Han M. Xu X. Dong J. (2020). The psychological impact of the COVID-19 epidemic on college students in China. *Psychiatry Res.* 287:112934. 10.1016/j.psychres.2020.112934 32229390 PMC7102633

[B5] Elmer T. Mepham K. Stadtfeld C. (2020). Students under lockdown: comparisons of students’ social networks and mental health before and during the COVID-19 crisis in Switzerland. *PLoS One* 15:e0236337. 10.1371/journal.pone.0236337 32702065 PMC7377438

[B6] Huang Y. Zhao N. (2020). Generalized anxiety disorder, depressive symptoms and sleep quality during COVID-19 outbreak in China: a web-based cross-sectional survey. *Psychiatry Res.* 288:112954. 10.1016/j.psychres.2020.112954 32325383 PMC7152913

[B7] Huckins J. Hedlund E. L. Rogers C. Nepal S. K. Wu J. Obuchi M. (2020). Mental health and behavior during the early phases of the COVID-19 pandemic: a longitudinal mobile smartphone and ecological momentary assessment study in college students. *J. Med. Internet Res.* 22:e20185. 10.2196/20185 PMC7301687 32519963

[B8] Liu N. Zhang F. Wei C. Jia Y. Shang Z. Sun L. (2020). Prevalence and predictors of PTSS during COVID-19 outbreak in China hardest-hit areas: gender differences matter. *Psychiatry Res.* 287:112921. 10.1016/j.psychres.2020.112921 32240896 PMC7102622

[B9] Odriozola-González P. Planchuelo-Gómez Á Irurtia M. J. de Luis-García R. (2020). Psychological effects of the COVID-19 outbreak and lockdown among students and workers of a Spanish university. *Psychiatry Res.* 290:113108. 10.1016/j.psychres.2020.113108 32450409 PMC7236679

[B10] Sahu P. (2020). Closure of universities due to coronavirus disease 2019 (COVID-19): impact on education and mental health of students and academic staff. *Cureus* 12:e7541. 10.7759/cureus.7541 32377489 PMC7198094

[B11] Satow L. (2012). *Stress- und Coping-Inventar (SCI): Testmanual und Normen*. Available online at: http://www.drsatow.de (accessed January 20, 2021).

[B12] Sønderskov K. M. Dinesen P. T. Santini Z. I. Østergaard S. D. (2020). The depressive state of Denmark during the COVID-19 pandemic. *Acta Neuropsychiatrica* 32 226–228. 10.1017/neu.2020.15 32319879 PMC7176490

[B13] Tambs K. Røysamb E. (2014). Selection of questions to short-form versions of original psychometric instruments in MoBa. *Nor. Epidemiol.* 24 195–201.

[B14] Wang C. Pan R. Wan X. Tan Y. Xu L. McIntyre R. S. (2020). A longitudinal study on the mental health of general population during the COVID-19 epidemic in China. *Brain Behav. Immun.* 87 40–48. 10.1016/j.bbi.2020.04.028 32298802 PMC7153528

[B15] Wang H. Xia Q. Xiong Z. Li Z. Xiang W. Yuan Y. (2020). The psychological distress and coping styles in the early stages of the 2019 coronavirus disease (COVID-19) epidemic in the general mainland Chinese population: a web-based survey. *PLoS One* 15:e0233410. 10.1371/journal.pone.0233410 32407409 PMC7224553

[B16] Wristen B. G. (2013). Depression and anxiety in university music students. Update: applications of research in music education. *SAGA J.* 31 20–27. 10.1177/8755123312473613

[B17] Xiong J. Lipsitz O. Nasri F. Lui L. M. Gill H. Phan L. (2020). Impact of COVID-19 pandemic on mental health in the general population: a systematic review. *J. Affect. Disord.* 277 55–64. 10.1016/j.jad.2020.08.001 32799105 PMC7413844

